# Working memory, negative affect and personal assets: How do they relate to mathematics and reading literacy?

**DOI:** 10.1371/journal.pone.0218921

**Published:** 2019-06-27

**Authors:** Enrica Donolato, David Giofrè, Irene C. Mammarella

**Affiliations:** 1 Department of Special Needs Education, University of Oslo, Oslo, Norway; 2 Department of Developmental and Social Psychology, University of Padova, Padova, Italy; 3 Department of Educational Sciences, University of Genova, Genova, Italy; University of St Andrews, UNITED KINGDOM

## Abstract

**Introduction:**

Research has recently focused on the relationships between working memory, negative affect (e.g., general anxiety, depressive symptoms) and personal assets (e.g., self-concept, academic and competence dimensions, and ego-resiliency), and their influence on mathematics and reading literacy. Although these variables have been amply explored, previous research has usually considered each of these aspects in isolation.

**Method:**

In the present study, 143 schoolchildren in sixth to eighth grade were tested on general anxiety, depressive symptoms, working memory, self-concept (academic and competence scales), ego-resiliency, and mathematics and reading literacy.

**Results:**

Variance partitioning showed that all predictors, i.e., working memory, negative affect (i.e., general anxiety and depressive symptoms), and personal assets (i.e., self-concept, academic and competence dimensions, and ego-resiliency) explained a unique and shared portion of the variance in mathematics and reading literacy.

**Conclusions:**

Our findings point to the importance of investigating the relationship between these factors. Underlying implications and directions for future research are discussed.

## Introduction

Academic success is a key aspect of a child’s life and development. Schoolchildren are required to achieve goals that are of fundamental importance to their future employment, income, and health status in adult life [1–3]. A great deal of literature has focused on various aspects relating to educational and developmental outcomes. For example, several studies examined individual factors related to academic success, such as motivation [4–6], implicit theories [7,8], goal-directed effort [9], and academic emotions [10,11]. Among such factors, general anxiety and depressive symptoms (called ‘negative affect’ from here on) were found to be associated with lower academic achievement [12,13]. On the other hand, self-concept and ego-resiliency (called personal assets from here on) seem to support children in their academic life [14–16]. Previous research also showed that working memory (WM) is one of the most important factors involved in academic achievement [17–19]. Research has yet to consider the unique contribution of all these variables to school-aged children’s academic performance, however. To fill this gap, the present study aims to examine the unique and shared contribution of negative affect, personal assets and WM in two key academic domains relating to mathematics and reading literacy.

### Negative affect, personal assets and academic achievement

The effects of general anxiety and depressive symptoms on academic achievement have been widely investigated. General anxiety can be defined as an individual's tendency to feel anxious about everyday situations, which may involve physiological anxiety, worry and social anxiety [20,21]. Depressive symptoms refer to low mood, intrusive ruminative thoughts, loss of interest and social withdrawal [22,23]. Current research on the prevalence of these emotional problems suggests that approximately 2.5% to 5% of children and adolescents meet the criteria for an anxiety disorder at some point [24–27], and that 2.8% to 8% of children and adolescents suffer from a depressive disorder [28,29]. As for the relationship between general anxiety and depressive symptoms, these two aspects frequently co-occur in childhood [30], and individuals suffering from these emotional problems often experience academic difficulties such as lower grades and graduation rates [13,31–33]. A recent meta-analysis confirmed that general anxiety and depressive symptoms tend to be negatively associated with school attainment [12]. It should be noted, however, that most of the previous research focused on clinical samples, while the few studies on typically-developing children produced inconsistent findings [34,35].

Alongside general anxiety and depressive symptoms, other factors related to school attainment include personal assets, for instance. Personal assets are features that help children to foster competence and promote their successful development in the personal, social and academic domains [36,37]. Among the various personal assets, self-concept and ego-resiliency can support children in different aspects of everyday life, including their academic performance [38–40]. Self-concept can be defined as a multidimensional and context-dependent learned behavioral pattern, based on personal assessments of one’s own past behavior and experiences, that influence an individual’s present and future behavior [41]. Ego-resiliency can be defined as a set of personal characteristics that help an individual to adapt to the environment, and recover quickly from everyday life challenges and difficulties [42,43]. A more comprehensive elaboration of the ego-resiliency construct suggests that ego-resilient people can show general resourcefulness, strength of character, and flexibility of functioning that enable them to adapt to, and recover from internal and environmental stressors, and everyday difficulties more quickly [44]. For example, ego-resilient individuals tend to use problem-solving abilities and be more persistent in seeking to achieve their goals [45,46]. Previous studies have shown that self-concept and ego-resiliency are associated with academic achievement [14–16,47,48]. A meta-analysis found a significant positive association (albeit small in magnitude) between self-concept and academic achievement, even after controlling for the initial level of achievement [15]. The effect of self-concept is also significant after controlling for children’s cognitive abilities [47]. In a similar vein, ego-resiliency has been found positively associated with academic performance in childhood and early adolescence [16,46], even taking cognitive and socioeconomic factors into account [49,50]. Overall, these results provide evidence of the role of negative affect and personal assets in students’ academic achievement, but there is still a paucity of research on how these personal assets take effect in specific academic domains related to mathematics and reading literacy. The vast majority of studies considered how negative affect and personal assets contribute to a single measure of academic achievement (i.e., school grades) [12,32,33,49,51], or in individuals with learning disabilities [52–55], or in terms of mathematical achievement [56–59]. Measures of reading literacy have been largely neglected, with only a few exceptions [46,47,60,61]. It is consequently still not clear whether the contributions of negative affect and personal assets are similar in explaining mathematics and reading literacy, or whether these variables could have a different influence on these two academic domains [62].

### Working memory, negative affect and academic achievement

Among several cognitive abilities, WM seems to be one of the most important to academic success [17,18]. Working memory is a limited-capacity system that enables information to be temporarily stored and manipulated [63,64]. The most classical theorization of WM is the tripartite model involving a *central executive system* responsible for controlling two subsidiary systems (i.e., the *phonological loop* and the *visuospatial sketchpad*) for verbal and visuospatial information [64,65]. Although this model has generated a broad consensus [64], other research has suggested a modality-independent model, in which WM is seen as a domain-general factor, without distinguishing between verbal and visuospatial components [66]. Research has shown that WM is an important facet of academic performance in reading comprehension [67,68] and mathematics achievement [69–71]. While there is a general agreement on this positive association between WM and academic achievement, it is only recently that researchers have started to examine the relationship between these aspects and symptoms of anxiety and/or depression.

Recent evidence indicates that anxiety and depressive symptoms could relate, at least in part, to WM. In the case of anxiety, some researchers found that worrying–i.e., cognitive elements of the experience of anxiety, such as negative expectations of oneself [72]–gives rise to task-irrelevant cognitions that reduce the WM available to complete a task [21,73]. A recent meta-analysis found a moderate negative association between anxiety and WM [35]. Similarly, depressive symptoms are characterized by intrusive ruminative thoughts that can interfere with cognitive processes, again reducing the ability to apply cognitive resources to complex cognitive tasks [23,74]. Although previous evidence seemed to suggest that anxiety and depressive symptoms can relate to poor WM, how these aspects relate to students’ academic achievement has not been thoroughly investigated [35].

### The present study

The aim of the present study was to assess the specific contributions of negative affect (i.e., general anxiety and depressive symptoms), WM and personal assets (i.e., self-concept, academic and competence dimensions, and ego-resiliency) to mathematics and reading literacy in middle-school students. We opted to consider this particular age group because early adolescence is an important stage of life when individuals are more likely to face emotional difficulties and a decrease in self-concept [37,75]. Confirmatory factor analyses (CFAs) were run to test the structure of the variables considered. Then, given our focus on clarifying the specific contributions of these factors, we conducted a variance decomposition analysis, examining the unique and shared contribution of negative affect (i.e., general anxiety and depressive symptoms), WM and personal assets (i.e., self-concept, academic and competence scales, and ego-resiliency) to mathematics and reading literacy. As regards negative affect and personal assets, these variables were expected to explain a unique portion of variance in both mathematics and reading literacy, even after accounting for WM [35,46,47,50]. As for WM, this ability was expected to explain a large and consistent, unique variance in both mathematics and reading literacy [67–70]. Research has also shown that specific emotional aspects (e.g., mathematics anxiety) relate to mathematical performance [56,58,59], while few studies have been conducted on reading literacy [60,76]. Due to the paucity of studies directly comparing mathematics with reading literacy, we assumed that the overall variance explained by negative affect and personal assets would be consistent for both academic domains, while possible differences might be related to WM (see [47]).

## Materials and methods

### Sample

The study involved 144 schoolchildren in grades 6 and 8. Using case-wise deletion with the Mahalanobis distance, one child was found to be a multivariate outlier (D > 49) and was excluded from the analyses, so the final sample included 143 children (females = 50%, age_months_ = 150.18, SD = 13.53) in grades 6 (n = 65, age_months_ = 136.75, *SD* = 6.13) and 8 (n = 78, age_months_ = 161.37, SD = 5.21). The children came from middle-class families and were attending schools in an urban area of north-east Italy. All the students tested were fluent in Italian. Children with special educational needs, intellectual disabilities, or neurological or genetic disorders were not considered. The study was approved by the Ethics Committee of the University of Padova, Italy. Parents’ written consent and children’s oral assent were obtained before testing.

### Materials

#### Negative affect

*The Revised Children’s Manifest Anxiety Scale*: *Second Edition* (RCMAS-2 [20]) is a self-report questionnaire used to identify general anxiety in children and adolescents. It consists of 40 items requiring a yes or no answer, such that higher scores indicate greater general anxiety. The self-report provides scores on worries (e.g., *“I feel nervous when things don’t go as I want”*), physiological anxiety (e.g., *“I often have stomach-ache”*) and social anxiety (e.g., *“I am worried that my classmates could make fun of me”*). The validated Italian version of the tool was used for the present study. As reported in the manual, the RCMAS-2 shows a good internal consistency for the subscales concerning worries (Cronbach’s α = .86), physiological anxiety (Cronbach’s α = .75), and social anxiety (Cronbach’s α = .80) [20].

The *Children’s Depression Inventory* (CDI; [77]) is a questionnaire for identifying relevant depressive symptoms in children and adolescents. This self-report consists of 27 items comprising three different statements (e.g., “*I am sometimes sad”–“I am often sad”–“I am always sad”*). For each item, respondents are asked to mark the sentence that best describes how they have been feeling and thinking during the preceding 2 weeks, choosing one of the three options. Each item is associated with a score based on severity, such that higher scores indicate more severe depressive symptoms. As reported in the manual, the CDI shows a good internal consistency for school-aged children (Cronbach’s α = .87) [77].

The *Questionnaire for the Assessment of Psychopathology in Adolescence* (Q-PAD; [78]) is a self-report questionnaire for assessing behavioral and emotional problems in children and adolescents. The *depression* scale was used for the present study; it identifies subclinical manifestations (i.e., not necessarily of clinical relevance) of sadness, boredom and melancholy linked to some degree of depression (e.g., *“Recently I have felt sad or melancholy most of the time”*). There are 8 items scored on a 4-point Likert scale ranging between 1 (“not describing my situation at all”) and 4 (“absolutely describing my situation”), such that higher scores indicate worse depressive symptoms. The manual reports a good internal consistency (Cronbach’s α = .78) [78].

#### Personal assets

The *Ego-resiliency Scale* (ER [43,44]) is an inventory for detecting resilience as a personality trait linked to general resourcefulness, strength of character, and flexibility of functioning (e.g., “*I quickly recover from being startled*”). The questionnaire consists of 14 statements scored on a 4-point Likert scale from 1 (“does not apply at all”) to 4 (“applies very strongly”), such that higher scores suggest a greater ego-resiliency. For the present study, an Italian translation of the tool, adapted to make the words comprehensible and suitable for children, was used. The internal consistency of the questionnaire was found adequate in the sample involved in the present study (Cronbach’s α = .69).

*Multidimensional Self-Concept Scale* (MSC[79]) is a self-report tool for assessing self-concept in children and adolescents. For the present study, the *Academic* (SC-A) and the *Competence* (SC-C) subscales were used to assess how participants perceived themselves at school (i.e., *“Studying is difficult for me”*), and their ability to influence their environment, solve problems or achieve their goals (i.e., *“I have faith in myself”*), respectively. Each subscale consists of 25 statements scored on a 4-point Likert scale from 1 (“absolutely false”) to 4 (“absolutely true”), such that higher scores indicate a more positive self-perception in the corresponding subscales. As reported in the manual, the tool has a good internal consistency for both the Academic and the Competence subscales (Cronbach’s α = .91 and α = .87 respectively) [79].

#### Working memory tasks

Verbal WM. *Word span—Backward* (WS-B; [80]). In the WS-B, lists of words were presented orally at a rate of one item per second and respondents had to recall the words in reverse order. The test proceeded from the shortest list to the longest (containing from two to eight words). No time limit was set for recalling the words in reverse order. The score was the raw number of words accurately recalled in the correct order (min 0, max 70). When calculated for the sample of children considered in this study, the internal consistency was adequate (Cronbach’s alphas .75).

*Verbal dual tasks* (DT-V [81]). In the DT-V, lists of words of medium-high frequency were presented orally. Each word list included four words, one or more of which were animal nouns. The word lists were organized into increasingly long sets of lists, and respondents were asked to recall the last word of each list in their order of presentation (i.e., from two to six words in all). For each list, they were also asked to press the space bar whenever they heard an animal noun. For instance, the child might hear two lists of words (and therefore have two words to recall, in their order of presentation), followed by three lists of words (with three words to recall) and so on. The score corresponded to the raw number of words accurately recalled at the end of each set of lists (min 0, max 40). Calculated on our sample of children, the internal consistency was adequate (Cronbach’s α = .69).

*Listening span test* (LST [82]). In the LST, children listened to sentences arranged into sets comprising from two to five sentences. The first set contained two sentences, and the number of sentences gradually increased in subsequent sets. After hearing each sentence, respondents were asked to judge whether the sentence was true or false. Then, after each set of sentences, they were asked to recall the last word in each sentence, in order of presentation. The score corresponded to the raw number of words accurately recalled (min 0, max 28). Calculated on our sample of children, the internal consistency was good (Cronbach’s α = .83).

Visuospatial WM. *Matrices span*—*Backward* (MS-B [80]). In the MS-B, the children had to recall the location of black cells that appeared briefly (for one second) in a series of different positions on the screen. Then they were asked to use the mouse to click on the locations where they had previously seen a black cell, in the order in which they had appeared. The number of cells presented in each series ranged from two to eight. The target appeared and disappeared on a grid visible in the middle of the screen. There was no time limit for recalling the cells and the score was the number of cells accurately recalled in the right order (min 0, max 70). Calculated on our sample of children, the internal consistency was good (Cronbach’s α = .79).

*Visuospatial dual tasks* (DT-VS [83]). The DT-VS consisted in a number of two-dimensional 4 × 4 grids, in which seven of the sixteen cells (i.e., one row and one column) were colored in gray while the others were white. The task was administered in sets of three grids, in which a black dot appeared in one of the cells, then disappeared. Respondents were asked to press the space bar if the dot appeared in a gray cell, and at the same time to remember the position of the last dot (in the third grid in each set). The sets of grids were arranged into six series, each comprising from two to six dots (stimuli) to be remembered (e.g. two stimuli in the first sets and more in later sets). The score corresponded to the raw number of dot positions in the last grid of each set that were accurately recalled, in order of presentation (min 0, max 40). Calculated on the current sample of children, the internal consistency was good (Cronbach’s α = .82).

*Dot matrix task* (DOT, derived from [84]). For each trial in this task, children were presented with sets of matrix equations to verify (i.e., additions or subtractions with lines in a grid of dots). After each equation, a dot appeared in a 5 × 5 grid and respondents were asked to remember its position, so they had to verify the matrix equation and simultaneously remember the dot’s location. The sets of matrixes were presented in four series of increasing length so that the location of 2 to 5 dots needed to be remembered (with two dots in the first set). The score corresponded to the number of dot locations correctly recalled (min 0, max 28). Calculated on our sample of children, the internal consistency was good (Cronbach’s α = .74).

#### Mathematics and reading literacy

The *INVALSI* (Italian Institute for the Assessment of the Education System [85]) comprises a set of tests widely used all over Italy to assess academic achievement in *mathematics* and *reading literacy* on a national scale. There are appropriate versions of the INVALSI tests for each school grade. As regards *mathematics*, the INVALSI tests provide scores in four areas: *space and figures* (MATH-SF) includes geometry-related tasks; *numbers* (MATH-N) measures number operations; *relations and functions* (MATH-RF) involves solving equivalences or algebraic expressions; and *data and prediction* (MATH-DP) requires the calculation of simple statistics, such as the probability of an event, or means. The mathematics test takes 75 minutes to complete. In the sample of the present study it showed a good internal consistency (Cronbach’s α = .84 and .85 for grades 6 and 8). As for *reading literacy*, the INVALSI generates scores relating to reading comprehension and grammar. For reading comprehension (READ-RC), the children were shown two written texts, and asked to answer several multiple-choice or open-ended questions. For grammar (READ-G), they were asked to answer questions on Italian language spelling, morphology, and lexicon. The reading literacy test takes 75 minutes. It showed good psychometric properties in our sample (Cronbach’s α = .87 and .88, for grades 6 and 8).

### Procedure

Participants were tested in different stages: a) in a group session in their classroom, lasting approximately 1 hour, when the self-report measures were administered; b) in individual sessions lasting approximately 30 minutes in a quiet room away from the classroom, when their WM was assessed; and c) in two group sessions lasting 75 minutes each, to test mathematics and reading literacy. As in previous studies, the tasks included in each section were presented in a pseudo-randomized order [47,76]. During the first session (November-December), the children completed the SC-Academic scale [79], the SC-Competence scale [79], the CDI [77], the ER-89 [43,44], the Q-PAD [78] and the RCMAS-2 [20]. In the second session (February-March), they took the WM tests in the following order: 1) MS-B [80]; 2) DT-V[81]; 3) DOT [84]; 4) LST [82]; 5) WS-B[80]; 6) DT-VS [83]. The WM tasks were programmed with E-prime-2 and presented on a 15-inch touchscreen laptop. The partial credit score method was used for scoring purposes [86]. The INVALSI tests [85] on mathematics and reading literacy were proposed during the last two sessions (in May of the same school year). In each session, the children were allowed to take a few minutes to rest between the tasks as necessary. A previously-published article examined subsets of the same data [87], focusing mainly on the influence of verbal and visuospatial WM on mathematics and reading literacy, but none of the analyses presented in this study have already been reported.

### Data analysis

Analyses were performed using R statistical software [88]. The “lavaan” library was used to conduct the confirmatory factor analysis (CFA) [89]. Model fit was assessed using various indexes according to the criteria suggested by Hu and Bentler [90], including: the chi-square (χ^2^); the comparative fit index (CFI); the non-normed fit index (NNFI); the standardized root mean square residual (SRMR); and the root mean square error of approximation (RMSEA). Since different versions of the INVALSI tests were used for the different school grades, item response theory (IRT) scaling, with the two-parameter model (2PL), was adopted to make the results comparable (for the rationale, see [91]). Raw residuals were calculated for each variable, controlling for age in months. In the first set of analyses, we performed a CFA to explore the latent factor structure of our indicators. Then we performed a series of regression analyses, which were then used as the baseline for the variance partitioning analyses (for a similar procedure, see [92]).

## Results

### Descriptive statistics

Descriptive statistics and correlations are provided in Table 1.

**Table 1:** Correlations and descriptive statistics (means and standard deviations) after controlling for age in months.

	1	2	3	4	5	6	7	8	9	10	11	12	13	14	15	16	17	18	19	20
1. DT-VS	─																			
2. DOT	.61	─																		
3. WS-B	.46	.47	─																	
4. DT-V	.54	.45	.31	─																
5. LST	.44	.33	.22	.68	─															
6. MS-B	.34	.34	.23	.36	.34	─														
7. CDI	-.07	-.11	-.07	-.18	-.06	-.05	─													
8. Q-PAD	-.09	-.06	-.03	-.15	-.07	-.09	.75	─												
9. RCMAS-PA	-.08	-.07	-.08	-.19	-.12	.04	.69	.59	─											
10. RCMAS -WO	-.07	-.11	.03	-.11	.03	-.05	.51	.51	.47	─										
11. RCMAS -SO	-.08	-.12	.00	-.20	-.07	-.06	.64	.57	.53	.68	─									
12. SC-C	-.04	-.06	-.01	.03	-.03	-.04	-.52	-.49	-.43	-.3	-.39	─								
13. SC-A	.05	-.02	.02	.23	.13	.05	-.50	-.44	-.40	-.11	-.37	.67	─							
14. ER	-.01	-.07	-.11	.04	.00	-.04	-.37	-.22	-.24	-.14	-.30	.44	.43	─						
15. MATH-SF	.24	.24	.24	.26	.20	.24	-.17	-.15	-.19	-.07	-.18	.12	.20	.05	─					
16. MATH-N	.33	.26	.19	.33	.16	.20	-.24	-.22	-.28	-.20	-.36	.19	.33	.08	.39	─				
17. MATH-RF	.35	.36	.33	.39	.32	.31	-.12	-.13	-.10	-.09	-.17	.03	.16	.08	.42	.52	─			
18. MATH-DP	.32	.29	.28	.33	.14	.28	-.14	-.20	-.17	-.11	-.16	.20	.27	.12	.37	.52	.45	─		
19. READ-RC	.22	.32	.10	.37	.32	.39	-.15	-.25	-.14	-.19	-.26	.18	.32	.14	.30	.46	.51	.50	─	
20. READ-G	.22	.24	.18	.29	.21	.34	-.23	-.23	-.24	-.11	-.21	.03	.25	.21	.37	.41	.46	.51	.58	─
M	22.72	15.28	44.59	17.27	13.94	35.77	8.79	14.45	3.26	6.08	4.08	75.78	71.15	44.11	41.85	41.97	42.57	47.59	63.44	69.76
DS	7.35	5.16	9.29	6.64	5.51	5.77	6.86	5.21	2.69	3.67	2.65	7.56	8.29	4.48	17.42	19.67	21.51	23.2	16.7	20.15

*Note*. All coefficients ≥ .17 are significant at .05 level. Means and standard deviations are calculated as the proportion of correct answers, before controlling for age or converting into IRT scores. DT-VS = Visuospatial dual tasks; DOT = Dot matrix task; MS-B = Matrices Span Backward; DT-V = Verbal dual tasks; LST = Listening span test; WS-B = Word span backward; CDI = Children’s depression inventory; Q-PAD = Questionnaire for the assessment of psychopathology in adolescence, depression subscale; RCMAS = general anxiety scale; -PA = physiological subscale; -WO = worries subscale; -SO = social subscale; SC = Self-concept scale; -A = Academic subscale; -C = Competence subscale; ER = Ego-resiliency scale; MATH = Mathematics; -SF = space and figures; -N = numbers; -RF = relations and functions; -DP = data and prediction; READ = Reading literacy; -RC = reading comprehension; -G = grammar

### CFA models

In Model 01, we hypothesized the presence of six latent variables: verbal WM (WM-V), visuospatial WM (WM-VS), general anxiety (ANX), depressive symptoms (DEP), personal assets (PER), mathematics (MATH), and reading literacy (READ). The fit of the model proved adequate: *χ*^*2*^(149) = 213.55, *p* < .001, *RMSEA* = .055, *SRMR* = .059, *CFI* = .946, *NNFI* = .931. Notably, the correlation between depressive symptoms and anxiety was positive and very high (*r* = .90), which might mean that these two factors are barely distinguishable. Factor loadings and inter-factor correlations for the model (Model 01) are shown in Table 2.

**Table 2:** Factor loadings, inter-factor and residual correlations for the final model.

	WM-VS	WM-V	DEP	ANX	RES	MATH	READ
DT-VS	0.82**						
DOT	0.75**						
WS-B	0.58**						
DT-V		0.89**					
LST		0.75**					
MS-B		0.45**					
CDI			0.92**				
Q-PAD			0.82**				
RCMAS-PA				0.74**			
RCMAS-WO				0.70**			
RCMAS-SO				0.81**			
SC-C					0.80**		
SC-A					0.83**		
ER					0.53**		
MATH-SF						0.54**	
MATH-N						0.71**	
MATH-RF						0.71**	
MATH-DP						0.70**	
READ-RC							0.79**
READ-G							0.73**
Inter-factor correlation matrix							
WM-VS	1						
WM-V	0.70**	1					
DEP	-0.12	-0.18	1				
ANX	-0.13	-0.21*	0.90**	1			
PER	-0.02	0.15	-0.69**	-0.55**	1		
MATH	0.59**	0.53**	-0.28**	-0.35**	0.34**	1	
READ	0.39**	0.52**	-0.30**	-0.35**	0.35**	0.87**	1

*Note*.

* *p* < .05

** *p* < .01.

DT-VS = Visuospatial dual tasks; DOT = Dot matrix task; WS-B = Matrices Span Backward; DT-V = Verbal dual tasks; LST = Listening span test; WS-B = Word span backward; CDI = Children’s depression inventory; Q-PAD = Questionnaire for the assessment of psychopathology in adolescence, depression subscale; RCMAS = general anxiety scale; -PA = physiological subscale; -WO = worries subscale; -SO = social subscale; SC = Self-concept scale; -A = Academic subscale; -C = Competence subscale; ER = Ego-resiliency scale; MATH = Mathematics; -SF = space and figures; -N = numbers; -RF = relations and functions; -DP = data and prediction; READ = Reading literacy; -RC = reading comprehension; -G = grammar; WM-VS = Working memory visuospatial; WM-V = Working memory verbal; PER = Personal assets.

### Variance partitioning

**Fig 1:**
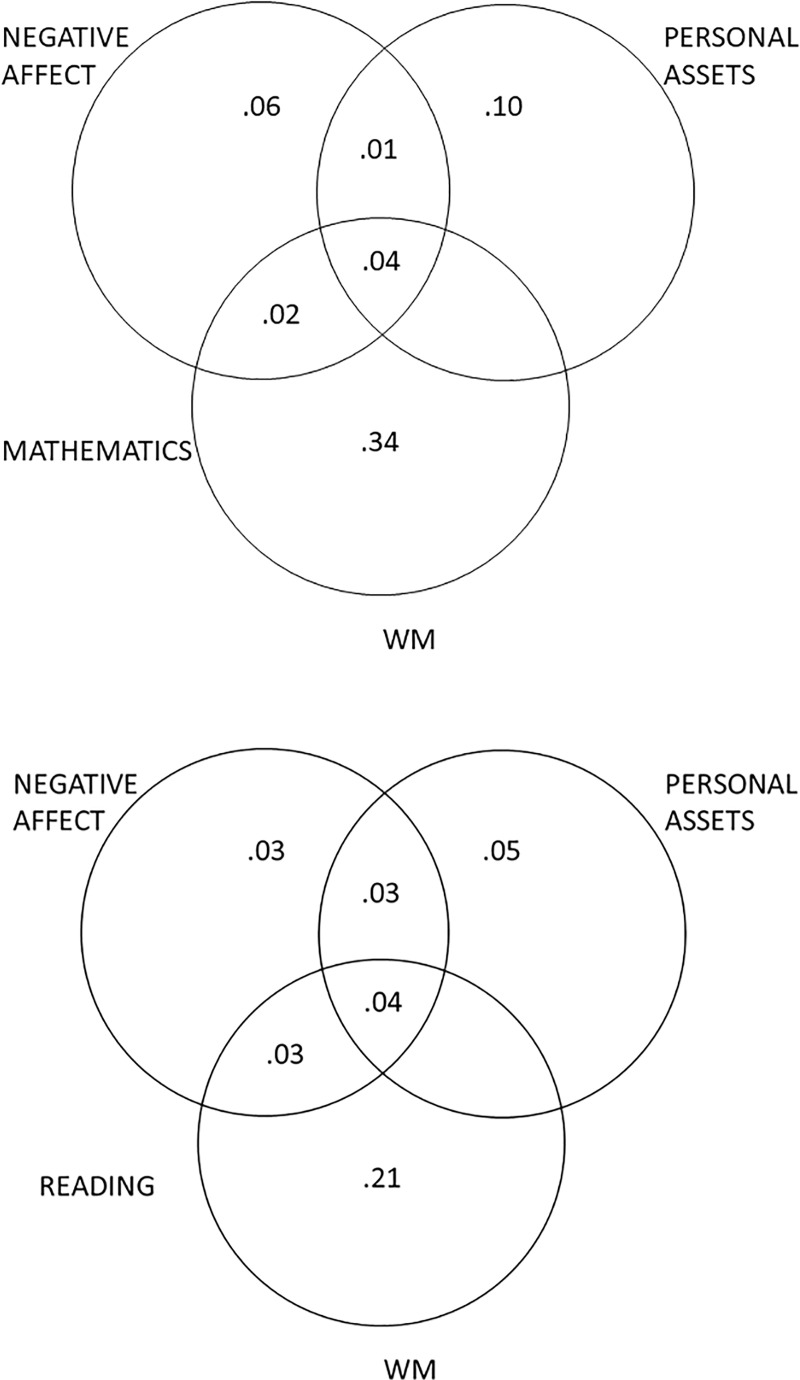
Variance partitioning for mathematics (top) and reading (bottom). Values ≤ 0 not shown.

To reduce the complexity of the analyses, we considered the overall role of WM, without distinguishing between its verbal and visuospatial components (see [92]), and the overall role of negative affect without distinguishing between depressive symptoms and anxiety. As concerns WM, its verbal and visuospatial aspects are distinguishable, but closely related [93]. The strong positive correlation (*r* = .70) between verbal and visuospatial WM components tends to produce multicollinearity problems, and this is particularly troublesome for the analyses that we wished to perform. As mentioned previously, anxiety and depressive symptoms were extremely closely related, so these variables were included in a single step. This decision is supported by previous literature suggesting that these aspects are empirically hardly distinguishable in children [30]. In this set of analyses, we used variance partitioning to examine the unique contribution of negative affect, WM and personal assets, and their combinations of shared variance, to mathematics and reading literacy. Several regression analyses were conducted (for a similar procedure, see [94]). As shown in Fig 1, the overall contributions to mathematics and reading literacy were quite similar, though the portion of explained variance was larger for the former (57%) than for the latter (39%). The personal assets variable explained a conspicuous portion of the unique variance in both mathematics and reading literacy (10% and 5%, respectively). The portion of the unique variance explained by the negative affect variable was somewhat smaller (6% for mathematics and 3% for reading literacy). WM tended to explain a larger portion of the unique variance in both the academic domains considered (34% for mathematics and 21% for reading literacy). Intriguingly, the variance shared by WM, personal assets and negative affect was not particularly high (i.e., 4% in both academic domains).

## Discussion

The present study adds to the burgeoning literature on the role of negative affect (i.e., general anxiety and depressive symptoms), personal assets (i.e., self-concept, academic and competence dimensions, and ego-resiliency), and WM on mathematics and reading literacy. We focused here on a sample of middle-school students. Unlike the vast majority of such studies, however, we estimated the unique and shared contributions of these variables to mathematics and reading literacy. In particular, negative affect and personal assets were expected to explain a unique portion of variance in both the academic domains considered, even after accounting for WM [35,46,48,50], while WM was expected to explain a large and consistent unique variance on mathematics and reading literacy [67–70]. Research has shown that specific emotional aspects (e.g. mathematics anxiety) relate to mathematical performance [56,58,59], while research on reading has been rather limited, so investigating both domains at the same time seems to be of particular interest [76].

Our results show that all the factors considered make some unique contribution to the academic domains investigated. Personal assets were found to explain a portion of the unique variance on both mathematics and reading literacy, a result that confirms previous research and provides evidence of the positive contribution of personal assets to mathematics and reading literacy, even after negative affect and WM have been taken into account [46,47,50]. Personal assets may provide an indication, for example, of how successfully children are studying and mastering their school work, supporting their achievement in both mathematics and reading literacy [14,31,38]. As for negative affect, this variable also explained a unique portion of the variance on both the academic domains considered. Our results are in line with previous findings of a role for negative affect in academic achievement [12]. More importantly, however, they suggest that negative affect can be related to reading literacy as well as mathematics. In particular, anxiety and depressive symptoms can generate a cognitive interference due to emotional distraction [35,95,96]. Our results also show that the contribution of negative affect cannot be subsumed by WM and personal assets: the unique contribution of negative affect was small, but not negligible. It is worth emphasizing that this is one of the first studies to have considered all these variables at once. As concerns WM, our results indicate that this cognitive factor explained a larger portion of the unique variance than either personal assets or negative affect, in both mathematics and reading literacy [67,68,70,71]. We also found that the overall variance in mathematics and reading literacy explained by negative affect, WM and personal assets together differed somewhat. This seems to be due to WM performance, however, which tends–like other higher cognitive functions–to be more strongly related to mathematics [47]. Thus, while negative affect and personal assets both seem to explain a (slightly different) unique portion of the variance in mathematics and reading literacy, further studies are needed to clarify whether general anxiety and depressive symptoms, or ego-resiliency and self-concept could add different, specific contributions to mathematics or reading literacy.

Although our study provides an important contribution to the literature, there are some limitations to consider in future research. For a start, the tests on academic achievement (i.e., the INVALSI), while very reliable, are used to assess children on the national curriculum in Italy, and are only available for grades 6 and 8 (which is why we focused only on these age groups). In addition, our moderate sample size prevented us from comparing our results across genders and school grades. Future studies should replicate our findings in a larger sample and age range, and examine to what extent negative affect, personal assets and academic performance tend to change across different school grades and in relation to gender. Girls seem to experience more anxiety and depression than boys [24,27,97,98]. Other studies that identified gender-related differences in school performance suggested that girls outperform boys at school throughout primary school and up to grade 8 [99]. The extent to which the relationship between emotional problems and academic performance differs between boys and girls is still unclear, however, and further research is needed [12]. Future studies should also examine the role of both general and specific forms of anxiety (e.g., mathematics anxiety, test anxiety) and their interplay with resilience in particular. As suggested by a recent study, different forms of anxiety may co-occur with high levels of general anxiety or academic anxiety (e.g., mathematics and test anxiety) [61,100]. This goes to show that it is important to examine both domain-general and domain-specific individual variables in order to elucidate their effects on academic performance. Finally, further research with a longitudinal design is needed to analyze the reciprocal influences of negative affect, personal assets, WM and academic achievement over time.

Our findings have some important implications. For a start, school-based intervention programs can be proposed for children with emotional difficulties as part of the formal school curriculum or after-school activities [101–103]. Prevention and intervention programs focusing on personal assets, such as self-concept and ego-resiliency, might be particularly useful to help children develop new skills and support them in achieving academic success [49,104,105]. Parents and teachers should help children to: a) build a positive overall and academic self-concept by reducing the link between test results and worthiness as a person; b) develop a positive approach to problem-solving in order to cope with difficulties and worries; and c) be aware that poor performance can be part of the learning process, and personal growth [104,106,107]. For example, social-emotional learning (SEL) programs have been found to foster the development of cognitive, affective, and behavioral competences, relationship skills, and responsible decision-making [108,109]. These competences could facilitate academic performance, as reflected in more positive social behavior, less emotional distress, and better test scores and grades [110,111].

To conclude, schoolchildren’s academic achievement seems to be related to several factors, including negative affect, personal assets and WM. The contribution of WM was found stronger than that of the other predictors, but both personal assets (to a larger extent) and negative affect (to a smaller extent) make a specific and shared contribution to mathematics and reading literacy. This finding points to the importance of considering cognitive and individual factors jointly in order to better understand schoolchildren’s academic success.

## References

[1] Fischbach A, Keller U, Preckel F, Brunner M. PISA proficiency scores predict educational outcomes. Learn Individ Differ. 2013;24:63–72.

[2] Mirowsky J, Ross CE. Education and self-rated health: Cumulative advantage and its rising importance. Res Aging. 2008;30(1):93–122.

[3] Roth PL, BeVier CA, Switzer FS, Schippmann JS. Meta-analyzing the relationship between grades and job performance. J Appl Psychol. 1996;81(5):548–56.

[4] Eccles JS, Wigfield A. Motivational beliefs, values, and goals. Annu Rev Psychol. 2002;1(53):109–32. 10.1146/annurev.psych.53.100901.135153 11752481

[5] Sutter-Brandenberger CC, Hagenauer G, Hascher T. Students’ self-determined motivation and negative emotions in mathematics in lower secondary education—Investigating reciprocal relations. Contemp Educ Psychol. 2018;55:166–75. Available from: 10.1016/j.cedpsych.2018.10.002

[6] Wigfield A, Eccles JS. Expectancy-value theory of achievement motivation. Contemp Educ Psychol. 2000;25(1):68–81. 10.1006/ceps.1999.1015 10620382

[7] Romero C, Master A, Dave Paunesku, Carol SD, Gross JJ. Academic and emotional functioning in middle school: The role of implicit theories. Emotion. 2014;14(2):227–34. 10.1037/a0035490 24512251

[8] Sisk VF, Burgoyne AP, Sun J, Butler JL, Macnamara BN. To What Extent and Under Which Circumstances Are Growth Mind-Sets Important to Academic Achievement? Two Meta-Analyses. Psychol Sci. 2018;29(4):549–71. 10.1177/0956797617739704 29505339

[9] Duckworth AL. Self-Regulation and School Success. Self-Regulation Auton Soc Dev Dimens Hum Conduct. 2013;208–30.

[10] Pekrun R, Lichtenfeld S, Marsh HW, Murayama K, Goetz T. Achievement Emotions and Academic Performance: Longitudinal Models of Reciprocal Effects. Child Dev. 2017;88(5):1653–70. 10.1111/cdev.12704 28176309

[11] Putwain DW, Becker S, Symes W, Pekrun R. Reciprocal relations between students’ academic enjoyment, boredom, and achievement over time. Learn Instr. 2018;54:73–81. Available from: 10.1016/j.learninstruc.2017.08.004

[12] Riglin L, Petrides K V., Frederickson N, Rice F. The relationship between emotional problems and subsequent school attainment: A meta-analysis. J Adolesc. 2014;37(4):335–46. 10.1016/j.adolescence.2014.02.010 24793380

[13] Seipp B. Anxiety and academic performance: A meta-analysis of findings. Anxiety Res. 1991;4(1):27–41.

[14] Martin AJ, Marsh HW. Academic resilience and its psychological and educational correlates: A construct validity approach. Psychol Sch. 2006;43(3):267–81.

[15] Valentine JC, DuBois DL, Cooper H. The Relation Between Self-Beliefs and Academic Achievement: A Meta-Analytic Review. Educ Psychol. 2004;39(2):111–33.

[16] Swanson J, Valiente C, Lemery-Chalfant K, O’Brien TC. Predicting early adolescents’ academic achievement, social competence, and physical health from parenting, ego resilience, and engagement coping. J Early Adolesc. 2011;31(4):548–76.

[17] St Clair-Thompson HL, Gathercole SE. Executive functions and achievements in school: Shifting, updating, inhibition, and working memory. Q J Exp Psychol. 2006;59(4):745–59. 10.1080/17470210500162854 16707360

[18] Gathercole SE, Pickering SJ, Knight C, Stegmann Z. Working memory skills and educational attainment: Evidence from national curriculum assessments at 7 and 14 years of age. Appl Cogn Psychol. 2004;18(1):1–16.

[19] Bull R., Scerif G. Executive Functioning as a Predictor of Children’s Mathematics Ability: Inhibition, Switching, and Working Memory. Dev Neuropsychol. 2001;19(3):273–93. 10.1207/S15326942DN1903_3 11758669

[20] Reynolds C, Richmond B. Revised Children’s Manifest Anxiety Scale: Second Edition (RCMAS-2). Giunti O.S., editor. Florence, Italy; 2012.

[21] Eysenck M. W., Calvo MG. Anxiety and Performance: The Processing Efficiency Theory. Cogn Emot. 1992;6:409–34.

[22] Kirkcaldy B., Siefen G. Depression, anxiety and self-image among children and adolescents. Sch Psychol Int. 1998;19(2):135–49.

[23] Nolen-Hoeksema S. The role of rumination in depressive disorders and mixed anxiety/depressive symptoms. J Abnorm Psychol. 2000;109(3):504–11. 11016119

[24] Costello EJ, Mustillo S, Erkanli A, Keeler G, Angold A. Prevalence and development of psychiatric disorders in childhood and adolescence. Arch Gen Psychiatry. 2003;60(8):837–844. 10.1001/archpsyc.60.8.837 12912767

[25] Ford T, Goodman R, Meltzer H. The British child and adolescent mental health survey 1999: The prevalence of DSM-IV disorders. J Am Acad Child Adolesc Psychiatry. 2003;42(10):1203–11. Available from: 10.1097/00004583-200310000-00011 14560170

[26] Lewinsohn PM, Hops H, Roberts RE, Seeley JR, Andrews JA. Adolescent psychopathology: I. Prevalence and incidence of depression and other DSM-III-R disorders in high school students. J Abnorm Psychol. 1993;102(1):133–44. 8436689 10.1037//0021-843x.102.1.133

[27] Lewinsohn PM, Zinbarg R, Seeley JR, Lewinsohn M, Sack WH. Lifetime comorbidity among anxiety disorders and between anxiety disorders and other mental disorders in adolescents. J Anxiety Disord. 1997;11(4):377–94. 9276783 10.1016/s0887-6185(97)00017-0

[28] Costello EJ, Erkanli A, Angold A. Is there an epidemic of child or adolescent depression? J Child Psychol Psychiatry Allied Discip. 2006;47(12):1263–71. 10.1111/j.1469-7610.2006.01682.x 17176381

[29] Lewinsohn PM, Clarke GN, Seeley JR, Rohde P. Major Depression in Community Adolescents: Age at Onset, Episode Duration, and Time to Recurrence. J Am Acad Child Adolesc Psychiatry. 1994;33(6):809–18. Available from: 10.1097/00004583-199407000-00006 7598758

[30] Seligman LD, Ollendick TH. Comorbidity of Anxiety and Depression in Children and Adolescents: An Integrative Review. Clin Child Fam Psychol Rev. 1998;1(2):125–44. 11324302 10.1023/a:1021887712873

[31] Ansary NS, Mcmahon TJ, Luthar SS. Socioeconomic Context and Emotional-Behavioral Achievement Links: Concurrent and Prospective Associations Among Low- and High-Income Youth. J Res Adolesc. 2012;22(1):14–30. 10.1111/j.1532-7795.2011.00747.x 23129975 PMC3488273

[32] Fröjd SA, Nissinen ES, Pelkonen MUI, Marttunen MJ, Koivisto AM, Kaltiala-Heino R. Depression and school performance in middle adolescent boys and girls. J Adolesc. 2008;31(4):485–98. 10.1016/j.adolescence.2007.08.006 17949806

[33] Verboom CE, Sijtsema JJ, Verhulst FC, Penninx BWJH, Ormel J. Longitudinal associations between depressive problems, academic performance, and social functioning in adolescent boys and girls. Dev Psychol. 2014;50(1):247–57. 10.1037/a0032547 23566082

[34] Cole DA, Martin JM, Powers B, Truglio R. Modeling causal relations between academic and social competence and depression. J Abnorm Psychol. 1996;105(2):258–70. 8723007 10.1037//0021-843x.105.2.258

[35] Moran TP. Anxiety and working memory capacity: A meta-analysis and narrative review. Psychol Bull. 2016;142(8):831–64. 10.1037/bul0000051 26963369

[36] Risk Dekovic M. and Protective Factors in the Development of Problem Behavior During Adolescence. J Youth Adolesc. 1999;28(6):667–85.

[37] Eccles JS. The development of children ages 6 to 14. Futur Child. 1999;9(2):30–44. 10646256

[38] Kuster F, Orth U, Meier LL. High Self-Esteem Prospectively Predicts Better Work Conditions and Outcomes. Soc Psychol Personal Sci. 2013;4(6):668–75.

[39] Masten AS. Ordinary magic: Resilience processes in development. Am Psychol. 2001;56(3):227–38. 11315249 10.1037//0003-066x.56.3.227

[40] Orth U, Robins RW, Widaman KF. Life-span development of self-esteem and its effects on important life outcomes. J Pers Soc Psychol. 2012;102(6):1271–88. 10.1037/a0025558 21942279

[41] Bracken BA. Handbook of self-concept: Developmental, social, and clinical considerations John Wiley & Sons, editor. Oxford, England; 1996.

[42] Becker BELSS. Social–Emotional Factors Affecting Achievement Outcomes Among Disadvantaged Students: Closing the Achievement Gap. Educ Psychol. 2012;37(4):197–214. 10.1207/S15326985EP3704_1 PMC3523355 23255834

[43] Block J, Kremen A. IQ and ego-resiliency: Clarifying their conceptual and empirical linkage and separateness. J Pers Soc Psychol. 1996;70(2):349–61. 8636887 10.1037//0022-3514.70.2.349

[44] Block, J. H., & Block J. The role of ego-control and ego resiliency in the organization of behavior. Hillsdale,. Erlbaum, editor. In W. A. Collins (Ed.), Minnesota symposium on child psychology. 1980.

[45] Bursik K, Martin TA. Ego development and adolescent academic achievement. J Res Adolesc. 2006;16(1):1–18.

[46] Liew J, Cao Q, Hughes JN, Deutz MHF. Academic Resilience Despite Early Academic Adversity: A Three-Wave Longitudinal Study on Regulation-Related Resiliency, Interpersonal Relationships, and Achievement in First to Third Grade. Early Educ Dev. 2018;29(5):762–79. 10.1080/10409289.2018.1429766 30197488 PMC6125773

[47] Giofrè D, Borella E, Mammarella IC. The relationship between intelligence, working memory, academic self-esteem, and academic achievement. J Cogn Psychol. 2017;29(6):731–47.

[48] Marsh HW, Trautwein U, Ludtke O, Koller O, Baumert J. Academic Self-Concept, Interest, Grades, and Standardized Test Scores: Reciprocal Effects Models of Causal Ordering. Child Dev. 2005;76(2):397–416. 10.1111/j.1467-8624.2005.00853.x 15784090

[49] Alessandri G, Zuffianò A, Eisenberg N, Pastorelli C. The Role of Ego-Resiliency as Mediator of the Longitudinal Relationship between Family Socio-Economic Status and School Grades. J Youth Adolesc. 2017;46(10):2157–68. 10.1007/s10964-017-0691-7 28540522

[50] Kwok O man, Hughes JN, Luo W. Role of resilient personality on lower achieving first grade students’ current and future achievement. J Sch Psychol. 2007;45(1):61–82. 18084626 10.1016/j.jsp.2006.07.002 PMC2140003

[51] Weidman AC, Augustine AA, Murayama K, Elliot AJ. Internalizing symptomatology and academic achievement: Bi-directional prospective relations in adolescence. J Res Pers. 2015;58:106–14. Available from: 10.1016/j.jrp.2015.07.005

[52] Mammarella IC, Ghisi M, Bomba M, Bottesi G, Caviola S, Broggi F, et al Anxiety and depression in children with nonverbal learning disabilities, reading disabilities, or typical development. J Learn Disabil. 2016;49(2):130–9. 10.1177/0022219414529336 24733818

[53] Snowling MJ, Muter V, Carroll J. Children at family risk of dyslexia: A follow-up in early adolescence. J Child Psychol Psychiatry Allied Discip. 2007;48(6):609–18. 10.1111/j.1469-7610.2006.01725.x 17537077

[54] Nelson JM, Harwood H. Learning disabilities and anxiety: A meta-analysis. J Learn Disabil. 2011;44(1):3–17. 10.1177/0022219409359939 20375288

[55] Graefen J, Kohn J, Wyschkon A, Esser G. Internalizing problems in children and adolescents with math disability. Zeitschrift fur Psychol / J Psychol. 2015;223(2):93–101.

[56] Hembree R. The Nature, Effects, and Relief of Mathematics Anxiety. J Res Math Educ. 1990;21(1):33–46.

[57] Justicia-Galiano MJ, Martín-Puga ME, Linares R, Pelegrina S. Math anxiety and math performance in children: The mediating roles of working memory and math self-concept. Br J Educ Psychol. 2017;87(4):573–89. 10.1111/bjep.12165 28561304

[58] Ma X. A Meta-Analysis of the Relationship between Anxiety toward Mathematics and Achievement in Mathematics. J Res Math Educ. 1999;30(5):520–40.

[59] Wu SS, Barth M, Amin H, Malcarne V, Menon V. Math anxiety in second and third graders and its relation to mathematics achievement. Front Psychol. 2012;3:1–11. 10.3389/fpsyg.2012.00001 22701105 PMC3369194

[60] Ackerman BP, Izard CE, Kobak R, Brown ED, Smith C. Relation between reading problems and internalizing behavior in school for preadolescent children from economically disadvantaged families. Child Dev. 2007;78(2):581–96. 10.1111/j.1467-8624.2007.01015.x 17381791

[61] Carey E, Devine A, Hill F, Szucs D. Differentiating anxiety forms and their role in academic performance from primary to secondary school. PLoS One. 2017;12(3):1–20. 10.1371/journal.pone.0174418 PMC5370099 28350857

[62] Mammarella IC, Caviola S, Dowker A. Mathematics Anxiety What is Known and What is still to be Understood. London: Routledge; 2019.

[63] Baddeley A. Working memory: Looking back and looking forward. Nat Rev Neurosci. 2003;4(10):829–39. 10.1038/nrn1201 14523382

[64] Baddeley A, Baddeley A. Exploring the Central Executive Exploring the Central Executive. Q J Exp Psychol. 1996;49(1):5–28.

[65] Baddeley AD. Working memory: theories, models, and controversies. Annu Rev Psychol. 2012;63(1):1–29. 21961947 10.1146/annurev-psych-120710-100422

[66] Kane MJ, Tuholski SW, Hambrick DZ, Wilhelm O, Payne TW, Engle RW. The generality of working memory capacity: A latent-variable approach to verbal and visuospatial memory span and reasoning. J Exp Psychol Gen. 2004;133(2):189–217. 10.1037/0096-3445.133.2.189 15149250

[67] Borella E, Carretti B, Pelegrina S. The Specific Role of Inhibition in Reading Comprehension in Good and Poor Comprehenders. J Learn Disabil. 2010;43(6):541–52. 10.1177/0022219410371676 20606207

[68] Borella E, de Ribaupierre A. The role of working memory, inhibition, and processing speed in text comprehension in children. Learn Individ Differ. 2014;34:86–92.

[69] Friso-Van Den Bos I, Van Der Ven SHG, Kroesbergen EH, Van Luit JEH. Working memory and mathematics in primary school children: A meta-analysis. Educ Res Rev. 2013;10:29–44.

[70] Passolunghi MC, Mammarella IC, Altoè G. Cognitive abilities as precursors of the early acquisition of mathematical skills during first through second grades. Dev Neuropsychol. 2008;33(3):229–50. 10.1080/87565640801982320 18473198

[71] Mammarella IC, Caviola S, Giofrè D, Szűcs D. The underlying structure of visuospatial working memory in children with mathematical learning disability. Br J Dev Psychol. 2018;36(2):220–35. 10.1111/bjdp.12202 28833308

[72] Morris LW, Davis MA, Hutchings CH. Cognitive and emotional components of anxiety: Literature review and a revised worry-emotionality scale. J Educ Psychol. 1981;73(4):541–55. 7024371

[73] Derakshan N, Eysenck MW. Anxiety, processing efficiency, and cognitive performance: New developments from attentional control theory. Eur Psychol. 2009;14(2):168–76.

[74] Christopher G, MacDonald J. The impact of clinical depression on working memory. Cogn Neuropsychiatry. 2005;10(5):379–99. 10.1080/13546800444000128 16571468

[75] Steinberg L, Morris AS. Adolescent devolepmental. Annu Rev Psychol. 2001;52:83–110. 10.1146/annurev.psych.52.1.83 11148300

[76] Hill F, Mammarella IC, Devine A, Caviola S, Passolunghi MC, Szucs D. Maths anxiety in primary and secondary school students: Gender differences, developmental changes and anxiety specificity. Learn Individ Differ. 2016;48:45–53.

[77] Kovacs, M., Camuffo, M., Cerutti, R., Lucarelli, L., Mayer R. CDI Children’s Depression Inventory: questionario di autovalutazione. O.S Giunti, editor. Florence, Italy; 1998.

[78] Sica C., Chiri L. R., Favilli R., Marchetti I. Questionario per la Valutazione della Psicopatologia in Adolescenza (Q-PAD) Erickson, editor. Trento, Italy; 2011.

[79] Bracken BA. Multidimensional Self Concept Scale. Erickson, editor. Trento, Italy; 2003.

[80] Cornoldi C, Vecchi T. Visuo-spatial working memory and individual differences Hove, UK: Psychology Press; 2003.

[81] De Beni R., Palladino P., Pazzaglia F., Cornoldi C. Increases in intrusion errors and working memory deficit of poor comprehenders. Q J Exp Psychol. 1998;51:305–320. 10.1080/713755761 9621841

[82] Daneman M., Carpenter PA. Individual differences in working memory and reading. J Verbal Learning Verbal Behav. 1980;19:450–466.

[83] Mammarella I. C., Cornoldi C. Difficulties in the control of irrelevant visuospatial information in children with visuospatial learning disabilities. Acta Psychol (Amst). 2005;118:211–228. 15698821 10.1016/j.actpsy.2004.08.004

[84] Miyake A, Friedman NP, Rettinger DA, Shah P, Hegarty M. How are visuospatial working memory, executive functioning, and spatial abilities related? A latent-variable analysis. J Exp Psychol Gen. 2001;130(4):621–40. 10.1037/0096-3445.130.4.621 11757872

[85] INVALSI. Servizio nazionale di valutazione 2010–11 [National evaluation service 2010–11]. INVALSI, editor. Rome, Italy; 2011.

[86] Conway ARA, Kane MJ, Al CET. Working memory span tasks—A methodological review and user’s guide. 2005;12(5):769–86. 16523997 10.3758/bf03196772

[87] Giofrè D, Donolato E, Mammarella IC. The differential role of verbal and visuospatial working memory in mathematics and reading. Trends Neurosci Educ. 2018;12(2017):1–6.

[88] R Core Team. R: A language and environment for statistical computing Vienna, Austria: R Foundation for Statistical Computing; 2016.

[89] Rosseel Y. lavaan: An R Package for Structural Equation Modeling. J Stat Softw. 2012;48(2):1–36.

[90] Hu LT, Bentler PM. Cutoff criteria for fit indexes in covariance structure analysis: Conventional criteria versus new alternatives. Struct Equ Model. 1999;6(1):1–55.

[91] Cook L. L., Eignor DR. IRT equating methods. Educational measurement: Issues and practice. 1991;10(3):37–45.

[92] Giofrè D, Mammarella IC. The relationship between working memory and intelligence in children: Is the scoring procedure important? Intelligence. 2014;46(1):300–10.

[93] Giofrè D, Mammarella IC, Cornoldi C. The structure of working memory and how it relates to intelligence in children. Intelligence. 2013;41(5):396–406.

[94] Chuah YML, Maybery MT. Verbal and Spatial Short-Term Memory: Common Sources of Developmental Change? J Exp Child Psychol. 1999;73(1):7–44. 10.1006/jecp.1999.2493 10196073

[95] Borkovec TD, Roemer L. Perceived functions of worry among generalized anxiety disorder subjects: Distraction from more emotionally distressing topics? J Behav Ther Exp Psychiatry. 1995;26(1):25–30. 7642757 10.1016/0005-7916(94)00064-s

[96] Putwain DW, Connors L, Symes W. Do cognitive distortions mediate the test anxiety-examination performance relationship? Educ Psychol. 2010;30(1):11–26.

[97] Culberston FM. Depression and gender: An international review. Am Psychol. 1997;52(1):25–31. 9017929 10.1037//0003-066x.52.1.25

[98] Weissman MM, Bland RC, Canino GJ, Faravelli C, Greenwald S, Hwu HG, et al Cross-national epidemiology of major depression and bipolar disorder. J Am Med Assoc. 1996;276(4):293–9. 8656541

[99] Organization for Economic Cooperation and Development. PISA 2012 assessment and analytical framework: Mathematics, reading, science, problem solving and financial literacy. OECD, editor. 2013.

[100] Mammarella IC, Donolato E, Caviola S, Giofrè D. Anxiety profiles and protective factors: A latent profile analysis in children. Pers Individ Dif. 2018;124:201–8.

[101] Heinrichs N, Bertram H, Kuschel A, Hahlweg K. Parent recruitment and retention in a universal prevention program for child behavior and emotional problems: Barriers to research and program participation. Prev Sci. 2005;6(4):275–86. 10.1007/s11121-005-0006-1 16075192

[102] Higgins E, O’Sullivan S. “What Works”: systematic review of the “FRIENDS for Life” programme as a universal school-based intervention programme for the prevention of child and youth anxiety. Educ Psychol Pract. 2015;31(4):424–38. Available from: 10.1080/02667363.2015.1086977

[103] Werner-Seidler A, Perry Y, Calear AL, Newby JM, Christensen H. School-based depression and anxiety prevention programs for young people: A systematic review and meta-analysis. Clin Psychol Rev. 2017;51:30–47. Available from: 10.1016/j.cpr.2016.10.005 27821267

[104] Martin A. J., Marsh HW. Fear of failure: Friend or foe?. Aust Psychol. 2003;38(1):31–8.

[105] Rose H, Miller L, Martinez Y. “FRIENDS for Life”: The Results of a Resilience-Building, Anxiety-Prevention Program in a Canadian Elementary School. Prof Sch Couns. 2010;12(6):400–7.

[106] Martin AJ. Examining a multidimensional model of student motivation and engagement using a construct validation approach. Br J Educ Psychol. 2007;77(2):413–40. 17504555 10.1348/000709906X118036

[107] Morrison GM, Allen MR. Promoting student resilience in school contexts. Theory Pract. 2007;46(2):162–9.

[108] Denham SA, Brown C. “Plays nice with others”: Social-emotional learning and academic success. Early Educ Dev. 2010;21(5):652–80.

[109] Panayiotou M, Humphrey N, Wigelsworth M. An empirical basis for linking social and emotional learning to academic performance. Contemp Educ Psychol. 2019;56(1):193–204. Available from: 10.1016/j.cedpsych.2019.01.009

[110] Durlak JA, Weissberg RP, Dymnicki AB, Taylor RD, Schellinger KB. The Impact of Enhancing Students’ Social and Emotional Learning: A Meta-Analysis of School-Based Universal Interventions. Child Dev. 2011;82(1):405–32. 10.1111/j.1467-8624.2010.01564.x 21291449

[111] Greenberg MT, Weissberg RP, O’Brien MU, Zins JE, Fredericks L, Resnik H, et al Enhancing School-Based Prevention and Youth Development Through Coordinated Social, Emotional, and Academic Learning. Am Psychol. 2003;58(6–7):466–74. 12971193 10.1037/0003-066x.58.6-7.466

